# Digestive Ferments

**Published:** 1880-08

**Authors:** Wm. Roberts

**Affiliations:** Prof. Clinical Medicine, Etc.


### Selections.

### DIGESTIVE FERMENTS.

BY WM. ROBERTS, F. R. C. P., F. R. S., PROF. CLINICAL MEDICINE, ETC.

#### PEPSIN AND TRYPSIN—DIGESTION OF PROTEIDS.

VARIOUS kinds of albuminous or proteid substances are used by mankind as food. The most important of these are muscular flesh, the casein of milk, and egg-albumen from the animal kingdom; and gluten, albumen, and legumin from the vegetable kingdom.

Proteids are attacked by the digestive ferments at two points in the alimentary canal, by pepsin in the stomach, and by trypsin in the small intestine. Between these two acts of digestion there is a complete break in the duodenum, owing to the abrupt change of reaction from acid to alkaline which occurs at that point.

### PEPTOGENS.

I may here advert to a single view advanced by Schiff in regard to the production and secretion of pepsin and trypsin. Schiff found that when an insoluble aliment such as white of egg (or fibrin, or meat which had been deprived of its soluble portions) was introduced into the stomach of a fasting animal no pepsin was secreted, and the albumen remained undigested; but if with the albumen certain soluble aliments were introduced into the stomach, then pepsin was produced, and digestion immediately began.

To these substances, which had the power of provoking the formation and secretion of pepsin, Schiff gave the name of *peptogens*. Among the most effective peptogens were found to be solutions of dextrine, extract of meat (or soup), infusion of green peas, bread (which contains dextrine), gelatine and peptones. Solutions of grape-sugar, soluble starch, fat-emulsion, or gum, however, had no peptogenic effect; and milk and coffee had not much. Schiff further found that peptogenic substances were just as effective when they were injected into the blood, or into the cellular tissue, or introduced as enemata into the rectum, as when they were introduced directly into the stomach. On the other hand, when peptogens were injected into the small intestine their influence was not observed; their effect seemed to be annulled by some action of the mesenteric glands, or by some change induced in them in their passage along the thoracic duct.

On the ground of these experiments—and they were numerous and oft-repeated and gave constant and decisive results—he concluded that the absorption into the blood of these soluble aliments was a necessary preliminary of proteid digestion—that no pepsin or trypsin was secreted unless these substances existed beforehand in the blood. The first act, according to Schiff, of gastric digestion was the absorption from the constituents of a meal of these soluble peptogens by the veins of the stomach. On this followed immediately the secretion of pepsin and the commencement of digestion proper.

These views and experiments of Schiff have not been allowed to pass without challenge, but they have not yet been overturned. If they should be substantiated they will give, curiously enough, a scientific sanction to the prevailing custom of commencing dinner with soup.

### THE MILK CURDLING FERMENT.

You all know that one of the most striking properties of gastric juice is to curdle milk. This property is utilized on a large scale in the industrial art of making cheese. Rennet, which has been used for that purpose from remote antiquity, is simply an infusion of the fourth stomach of the calf in brine. The curdling of casein by rennet does not depend upon the acid of the gastric juice, for it takes place when the milk is neutral or even faintly alkaline. It has until lately been believed that this property was an inherent attribute of pepsin, but this opinion is no longer tenable. Brücke succeeded, by a process I need not particularize, in producing pepsin which had an energetic action on proteids, but which did not possess, except in the feeblest degree, the power of curdling milk. Mr. Benger also found that an extract of pig's stomach in saturated brine, while it possessed energetic action as a milk-curdler, had only feeble proteolytic powers. We must, therefore, regard the agent in gastric juice which curdles milk as a substance distinct from pepsin.

In the course of my experiments on pancreatic extract, I made the unexpected observation that the pancreas also contained an agent capable of curdling milk. I found this property in the pancreas of the pig, the sheep, the calf, the ox, and the fowl. In whatever way the extract of the gland was made, whatever solvent was used, this property of curdling milk was present in it; but the brine extract exceeded all others in curdling capacity. If a few drops of extract of pancreas be added to some warm milk in a test-tube, the milk becomes a solid coagulum in a few minutes. Some minutes later the whey begins to separate from the curd. In short, the action resembles exactly that of calf's rennet; and, so far as I know, you could make cheese with pancreatic rennet as perfectly as you can with gastric rennet. There is, however, not an absolute indentity of the two agents. I said just now that gastric rennet produced curdling in neutral and even in faintly alkaline milk; but if the alkali exceed a very small proportion ordinary rennet does not curdle milk. I found that an alkalescence exceeding that produced by one grain of bicarbonate of soda to an ounce of milk altogether prevented the milk being curdled by gastric rennet. But this is not so with pancreatic rennet. You may add two, three, or four grains of bicarbonate of soda to each ounce of milk and still the pancreatic rennet will induce curdling with undiminished energy. Milk is likewise curdled by pancreatic extract when quite neutral, and even when very faintly acid. Indeed, it appeared to me that a very faintly acid milk curdled more actively with pancreatic extract than neutral milk, but not so actively as alkaline milk.

That the curdling agent of the stomach and pancreas is a true ferment, and not some inorganic chemical agent, seems to be proved by the fact that boiling or even heating to 160° F. (70° C.) instantly destroys its power. I found, moreover, that, like other soluble ferments, it is precipitated, but not truly coagulated, by alcohol—for it recovers its solubility and activity when the alcohol is removed, even after a contact of several weeks.

What is the real function of the curdling ferment? Seeing its striking reaction with milk, one's first idea is that it must have something to do with the digestion of casein. But a little consideration shows that this idea is altogether improbable. Although all mammalia start life on a milk diet, milk does not form a part of the normal diet of any adult creature except man. Nor can its universal presence in the mammalian digestive organs be regarded as a vestigial phenomenon—a "memory" of the sucking phase of their existence—for the same curdling property is found in the stomach and pancreas of the fowl, which never at any period of its life fed on milk. Moreover, it may be doubted whether the ferment in question is the actual agent which curdles milk on its passage into the stomach; for the acid of the gastric juice, which also curdles milk, would probably be beforehand with it, inasmuch as its action is a good deal more prompt than that of the ferment. In the pancreatic digestion of milk, the occurrence of curdling has appeared to me to be a distinct hindrance to the process. Has this ferment any true digestive functions? I think this is quite open to doubt. Its action on milk is apparently akin to that of the fibrin-ferment on blood—and it may likewise have some kindred purpose; but what that purpose may be I am unable to conjecture.

### DIGESTION OF FAT.

Some observations made by Brücke promise to throw a fresh light on the digestion of fat. Brücke found that oils and fats which contained an admixture of free fatty acids—in other words, which were more or less rancid—were emulsified by a slight agitation with a weak solution of carbonate of soda. J. Gad extended these observations, and showed that even simple contact of a rancid oil with the alkaline solution was sufficient to effect a mechanical division of the oily matter. I have repeated these observations, and the results are certainly remarkable. The different behavior of two specimens of the same oil, one perfectly neutral and the other containing a little free fatty acid, is exceedingly striking. I have here before me two specimens of cod-liver oil—one of them is a fine and pure pale oil, such as is usually dispensed by the better class of chemists; the other is the brown oil sent out under the name of De Jongh. I put a few drops of each of these into these two beakers, and pour on them some of this solution, which contains two per cent. of bicarbonate of soda. The pale oil, you see, is not in the least emulsified; it rises to the top of the water in large clear globules. The brown oil, on the contrary, yields at once a milky emulsion. The pale oil is a neutral oil, and yields no acid to water when agitated with it—in other words, it is quite free from rancidity; but the brown oil when treated in the same way causes the water with which it is shaken to redden litmus paper. I was surprised to find that olive oil (salad oil), which appeared quite sweet, and had not the slightest taste or smell of rancidity, gave a milky emulsion with the soda solution. This oil did not yield any acid reaction to water when agitated therewith. Nevertheless, it evidently contained a little free fatty acid (probably oleic acid, which is insoluble in water, and therefore does not acidify water shaken up with it), for when a portion of this oil was washed with a strong solution of carbonate of soda, and then allowed to separate, the oil thus freed from acid no longer gave an emulsion with the weak soda solution. It would appear that an admixture of only a very small proportion of free fatty acid is sufficient to induce emulsification—a quantity so small as not to cause any appreciable rancidity to the sense of smell or taste. This specimen of almond oil is to all appearance perfectly sweet, but it communicates a rather sharp acid reaction to water shaken up with it, and it gives, as you see, a perfect emulsion with the soda solution.

The bearing of these observations on the digestion of fat is plain. When the contents of the stomach pass the pylorus they encounter the bile and pancreatic juice, which are alkaline, from the presence in them of carbonate of soda. So that the fatty ingredients of the chyme, if they only contain a small admixture of free fatty acids, are at once placed in favorable circumstances for the production of an emulsion without the help of any soluble ferment, the mere agitation of the contents of the bowel by the peristaltic action being sufficient to effect the purpose.—*London Lancet.*

